# Geology of the Early Arikareean Sharps Formation on the Pine Ridge Indian Reservation and Surrounding Areas of South Dakota and Nebraska

**DOI:** 10.1371/journal.pone.0047759

**Published:** 2012-10-23

**Authors:** Thomas H. McConnell, Joseph N. DiBenedetto

**Affiliations:** United States Forest Service, Price, Utah, United States of America; ETH, Switzerland

## Abstract

Based on geologic mapping, measured sections, and lithologic correlations, the local features of the upper and lower type areas of the Early Arikareean (30.8–20.6 million years ago) Sharps Formation are revised and correlated. The Sharps Formation above the basal Rockyford Member is divided into two members of distinct lithotypes. The upper 233 feet of massive siltstones and sandy siltstones is named the Gooseneck Road Member. The middle member, 161 feet of eolian volcaniclastic siltstones with fluvially reworked volcaniclastic lenses and sandy siltstone sheets, is named the Wolff Camp Member. An ashey zone at the base of the Sharps Formation is described and defined as the Rockyford Ash Zone (RAZ) in the same stratigraphic position as the Nonpareil Ash Zone (NPAZ) in Nebraska. Widespread marker beds of fresh water limestones at 130 feet above the base of the Sharps Formation and a widespread reddish-brown clayey siltstone at 165 feet above the base of the Sharps Formation are described. The Brown Siltstone Beds of Nebraska are shown to be a southern correlative of the Wolff Camp Member and the Rockyford Member of the Sharps Formation. Early attempts to correlate strata in the Great Plains were slow in developing. Recognition of the implications of the paleomagnetic and lithologic correlations of this paper will provide an added datum assisting researchers in future biostratigraphic studies. Based on similar lithologies, the Sharps Formation, currently assigned to the Arikaree Group, should be reassigned to the White River Group.

## Introduction

The Arikareean North American Land Mammal Age (30.8–20.6 million years ago) was defined by the Wood committee in 1941, but due to the noncontinuous nature of the stratigraphic column as known at that time, the temporal sequence of a part of the paleofauna was unrecognized. A post-Whitneyan and pre-Arikareean paleofauna later came to be recognized between the White River Group and the Arikareean Group.

The Wood committee defined the upper boundary of the Whitneyan Land Mammal Age as those taxa representative of the top of the Whitney Member of the Brule Formation in Nebraska. In 1941 these sediments were thought to be the uppermost sediments of the White River Group. The lower boundary of the Arikareean Land Mammal Age was defined as those taxa representative of the base of the Gering Formation in Nebraska. These sediments were thought to be the lowermost sediments of the Arikaree Group. Both boundaries were based on sections in Nebraska. There is a disconformity at the base of the Gering Formation [1], [2]. The temporal hiatus represented by the missing sediments was not taken into account when the original concepts of the Arikareean and Whitneyan Land Mammal Ages were developed. The Sharps Formation [3] and its included paleofaunas [4], [5] and the Brown Siltstone Beds [6] account for this time hiatus.

The Sharps Formation was initially assigned an early Miocene age, but it has more recently been shown to be of late Oligocene [7]. As a result, biostratigraphic studies until very recently, placed the Sharps Formation paleofauna too high in the composite biostratigraphic column. Evidence now shows that the earliest paleofaunas of the Sharps Formation are antecedent to the paleofauna of the lowest portion of the Gering Formation [7]. The paleofauna of the Sharps Formation appears to overlap both the Whitneyan and Arikareean Land Mammal ages suggesting that it is a key to unravelling some of the complex correlations of the stratigraphy of the Great Plains of North America.

The White River Badlands of South Dakota in North America are well known for the fossiliferous nature of the sediments. Some areas surrounding Badlands National Park in the White River badlands of South Dakota have sediments slightly younger than the Brule Formation sediments prevalent in the park. These younger sediments were undiscovered until 1960 and generally inaccessible since then because the major outcrops are on the Pine Ridge Indian Reservation. The White River Group sediments, including the Sharps Formation, were deposited during the time the grasses were developing on the plains of North America and herbivorous animals were adapting to utilize this new resource. The carnivores of course followed their prey onto the plains. The White River Group sediments give us a window to explore the impact of grasses on the paleofauna of the plains as the animals adapt over millions of years in this new niche.

Harksen *et al.* (1961) described the Sharps Formation and shortly thereafter, Macdonald followed with two monographs [4], [5] on the contained paleofauna. The Pine Ridge Indian Reservation contains more complete outcrops of the Sharps Formation than surrounding areas and only two paleontologists have been permitted to carry out geological research within the borders of the Pine Ridge Indian Reservation, Macdonald, and twenty years later, McConnell. The scientific community has had limited access to paleontological materials on the Pine Ridge Indian Reservation. Any collections of fossils on the reservation that was done before the permitting process, or later without permission, were deemed unethical. Collecting is now unlawful on tribal lands without written permission from the Lakota Sioux Tribal Council. Most scientific works regarding the Sharps Formation, paleofauna, and stratigraphy were based on supposition rather than scientific observation of materials on the Pine Ridge Indian Reservation.

The discoveries of datable ash layers containing index fossils has confirmed the Oligocene/Miocene boundary occurs within the Sharps Formation. The Whitneyan/Arikareean (North American Land Mammal Age (NALMA) boundary likewise occurs within the formation [8].

This paper is the first of a number of papers that will clarify and correlate the lithology and describe the paleofauna previously neither described nor correlated regionally.

### Previous Work

**Figure 1:**
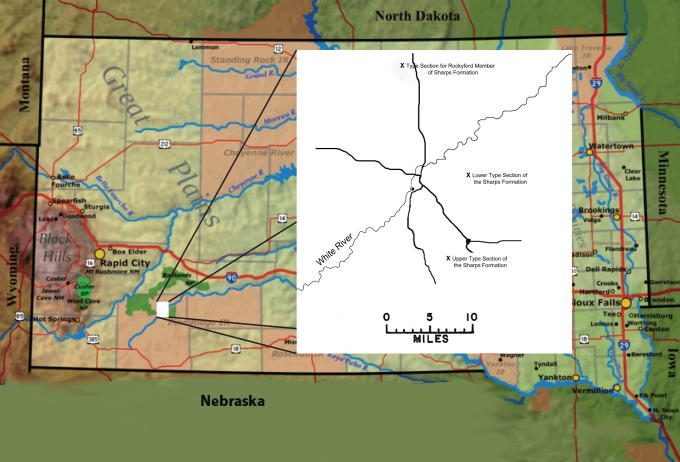
Research Area Showing Three Type Sections. Three locations separated by as much as twelve miles were used in the formal descriptions: one location for the type section of the Rockyford Member of the Sharps Formation, a second for the lower type section of the Sharps Formation, and a third for the upper type section of the Sharps Formation.

The original description of the Sharps Formation [3] described a strata occurring between the Monroe Creek Formation and the Brule Formation in South Dakota. The formation was assigned to the Arikaree Group, although the lithology was representative of White River sediments. The description of the basal Rockyford Member, the Rockyford Ash [9] immediately followed. Three different locations separated by as much as twelve miles were used in the formal descriptions (Figure 1): one location for the type section of the Rockyford Member, a second for the lower type section of the Sharps Formation, and a third for the upper type section of the Sharps Formation. The original use of geographically separated upper and lower type sections for the Sharps Formation [3] introduced an error in the interpretation of the total thickness of the formation.

Though the lithologies did not correlate, a large overlap of the upper and lower type section sediments was assumed, and the thickness of the Sharps Formation above the Rockyford Member was reported to be 349.3 feet.

The Rockyford Ash at Sheep Mountain, 10 miles north of the lower type section was selected as the type section of the Rockyford Member [9]. Using the Rockyford Ash as a reference, Nicknish and Macdonald (1962) reported a regional southeasterly 0.38% grade of approximately 20 feet per mile for the Sharps Formation.

Massive siltstone deposits dominate the upper 233 feet of the Sharps Formation. Intercalated within the lower half of the formation are numerous lenses of sandstone and siltstone, the highest of which occurs at approximately 165 feet above the base of the Sharps Formation.

## Analysis

### Regional Interpretation

**Figure 2:**
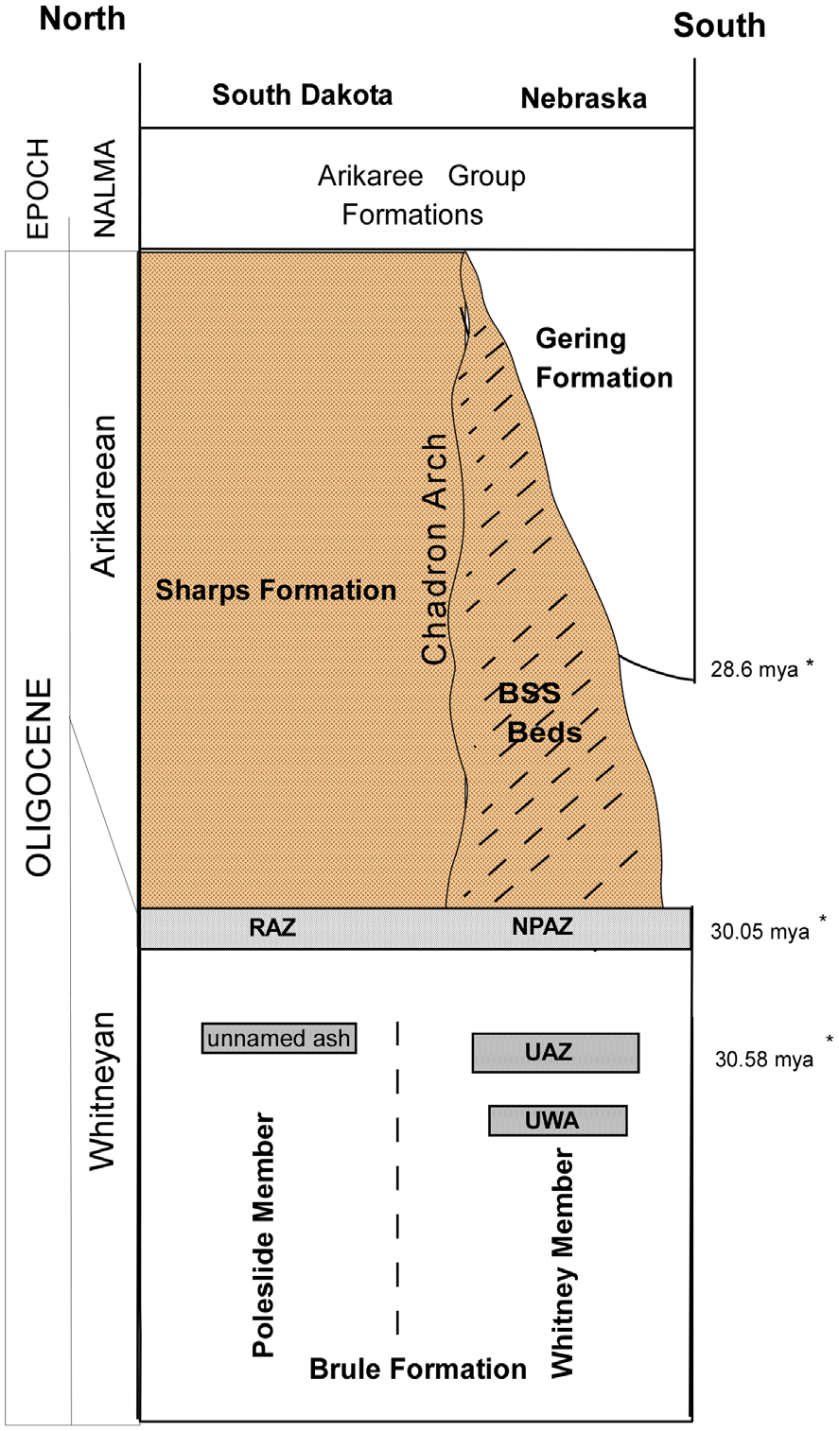
Stratigraphic Relationships of Some Great Plains Formations. Both the Poleslide Member, Brule Formation in South Dakota and the Whitney Member, Brule Formation in Nebraska are overlain conformably by lithologic bodies of approximately the same thickness: the Sharps Formation and the Brown Siltstone Beds [3], [7]. In the vicinity of the Chadron Arch, a thick tephra is identified at the base of both: the Rockyford Ash Zone (RAZ) in South Dakota [9] and the Nonpareil Ash Zone (NPAZ) in Nebraska [7]. The lower portions of the Brown Siltstone Beds and Sharps Formation are more stratified than their upper portions. The upper portions of each are massively bedded siltstone with intermittent, vertically oriented, spindle/potato-shaped nodules. The lithologies of the Brown Siltstone Beds and the Sharps Formation are similar. These data are consistent with the subsurface evidence of lateral continuity across the Chadron Arch [12]. The Nonpareil Ash Zone (NPAZ) of the Brown Siltstone Beds can be traced in the subsurface across the Chadron Arch. This ash complex northeast of the Chadron Arch is known as the Rockyford Ash Zone (RAZ) [9]. Swinehart, *et al.* (1985) provides direct subsurface evidence of the correlation of the NPAZ with the RAZ and the subsequent correlation of the Sharps Formation with the Brown Siltstone Beds.

Early attempts in the mid 1900’s to correlate strata in the Great Plains were slow in developing due to attempts to use the Gering Formation of Nebraska as a Paleogene datum for deposits of late Oligocene-early Miocene age [7], [10]. Schultz and Falkenbach interpreted the entire Sharps Formation of South Dakota as a facies and time correlative with the Gering Formation of Nebraska [10]. Tedford *et al.* [7] indicate the Sharps Formation is not a facies of the Gering Formation. Swisher and Prothero (1990) demonstrated the Gering Formation (*sensu stricto*) occupied only a limited area and a temporal range of one million years or less. The lithologies of the Gering Formation were unconformably deposited on sediments of the Whitney Member of the Brule Formation or Brown Siltstone Beds (Figure 2).

### Brown Siltstone Beds

Tedford *et al.* [7] recognized the Brown Siltstone Beds in Nebraska as stratigraphically correlative to the Sharps Formation in South Dakota. Later work [11] has supported this correlation. The Brown Siltstone Beds occur stratigraphically below the Gering Formation and above the Whitney Member of the Brule Formation. In some areas of Nebraska, the Gering Formation disconformably overlies the Whitney Member of the Brule Formation [1], [7] with no intervening Brown Siltstone Beds (Figure 2).

## Results and Discussion

### Regional Relationships

Although the Gering and Sharps Formations, and the Brown Siltstone Beds share a stratigraphic position immediately above the Brule Formation, they are not all of similar lithology. The Gering Formation is principally a sandstone [1], [7], whereas the Sharps Formation [3] and Brown Siltstone Beds [11] consist mainly of siltstones. Unfortunately there is no surface lateral continuity of the Gering Formation or Brown Siltstone Beds of Nebraska with the Sharps Formation of South Dakota as the regions were separated by the Chadron Arch, a regional structural feature trending NW-SE and oblique to the Nebraska-South Dakota state line [12]. However, the physical resemblances of the Brown Siltstone Beds and the Sharps Formation on each side of the arch suggest a similar source and well logs across the arch support this subsurface correlation [12].

The Brule Formation upward through the Gering Formation deposits in Nebraska correlate lithologically and paleomagnetically with rocks in South Dakota from Brule Formation upward through the Sharps Formation.

In Nebraska the Whitney Member of the Brule Formation is about 300 feet thick and consists of nodular volcaniclastic siltstone with local channel sandstones and mudstones [7] conformably overlying the Orella Member of the lower Brule Formation. Similarly in South Dakota, the Poleslide Member of the Brule Formation consisting of nodular volcaniclastic siltstone with local sandstones and mudstones is of similar thickness [13], and conformably overlies the Scenic Member of the Brule Formation. Magnetic polarity Chron C12R is contained within the Whitney Member of the Brule Formation and the Poleslide Member of the Brule Formation [14].

### Revised Stratigraphy

Both the Poleslide Member of the Brule Formation in South Dakota and the Whitney Member of the Brule Formation in Nebraska are overlain conformably by lithologic bodies of approximately the same thickness: the Sharps Formation and the Brown Siltstone Beds [3], [7]. In the vicinity of the Chadron Arch, a thick tephra is identified at the base of both: the Rockyford Ash Zone (RAZ) in South Dakota [9] and the Nonpareil Ash Zone (NPAZ) in Nebraska [7]. The lower portions of the Brown Siltstone Beds and Sharps Formation are more stratified than their upper portions. The upper portions of each are massively bedded siltstone with intermittent, vertically oriented, spindle/potato-shaped nodules. The lithologies of the Brown Siltstone Beds and the Sharps Formation are similar.

These data are consistent with the subsurface evidence of lateral continuity across the Chadron Arch [12]. The Nonpareil Ash Zone (NPAZ) of the Brown Siltstone Beds can be traced in the subsurface across the Chadron Arch. This ash complex northeast of the Chadron Arch is known as the Rockyford Ash Zone (RAZ) [9]. Swinehart, *et al.* (1985) provides direct subsurface evidence of the correlation of the NPAZ with the RAZ and the subsequent correlation of the Sharps Formation with the Brown Siltstone Beds.

Further confirmation of this correlation is provided by paleomagnetic data [15], [16]. Both the NPAZ and the RAZ were deposited near the base of magnetic polarity Chron C11N.

**Figure 3:**
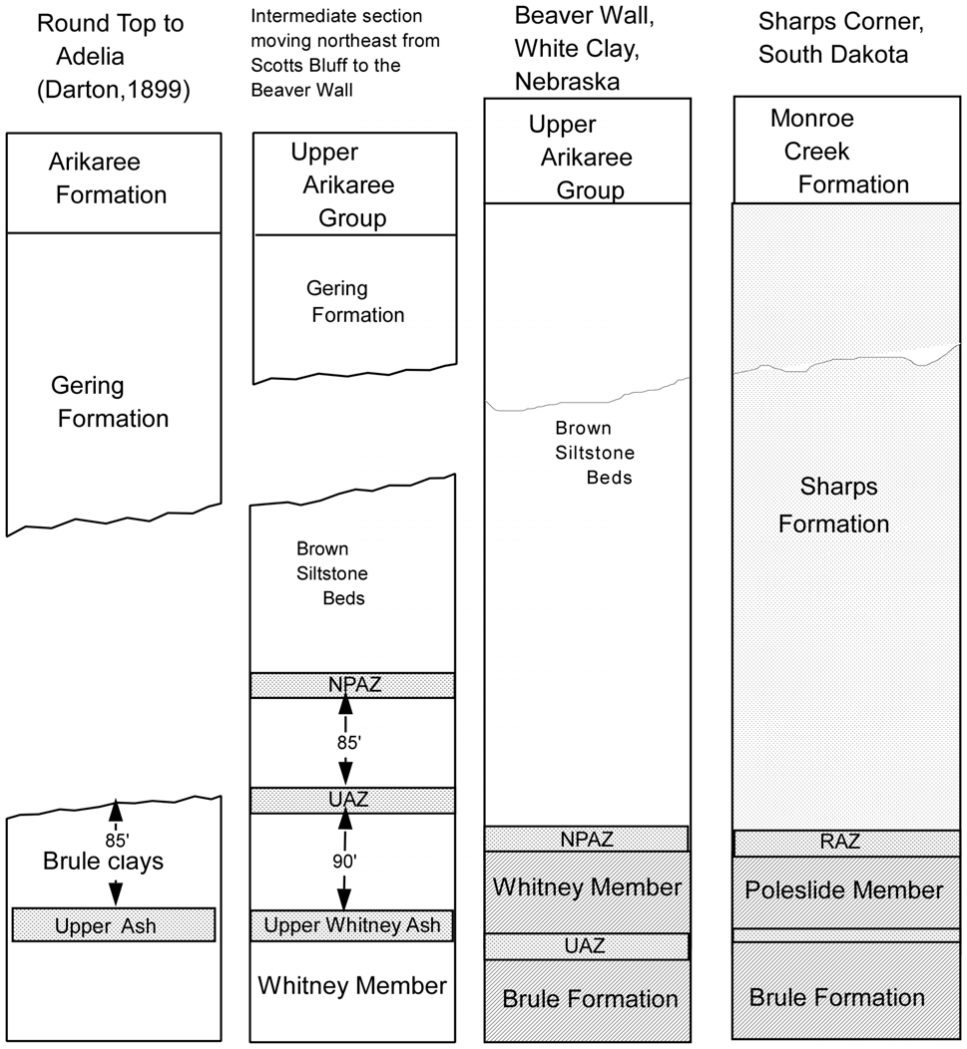
Stratigraphic Correlation of Sediments. At the southernmost extension of the Brown Siltstone Beds in Nebraska, these beds are quite thin and overlain by 200+ feet of the Gering Formation. The Brown Siltstone Beds are thicker northward. At the Round Top to Adelia section a complete section of the Gering Formation displays a basal disconformity with the underlying Whitney Member of the Brule Formation. There is evidence of an Upper Whitney Ash (UWA) and an Upper Ashy Zone (UAZ). Proceeding to the northeast, the Gering Formation pinches out, the Whitney ash beds disappear, and the NPAZ appears at the base of the Brown Siltstone [7] so that in the vicinity of the Chadron Arch and the town of White Clay, Nebraska, there is a fully developed section of the Brown Siltstone Beds with no overlying Gering Formation and no Whitney ashes. The NPAZ is a stratigraphic correlative of the RAZ, and the Brown Siltstone Beds in Nebraska occupy a position between the top of the Brule Formation and the Monroe Creek Formation identical to the Sharps Formation in South Dakota. Where the Gering Formation occurs in Nebraska, it disconformably overlies either Brule Formation sediments or the Brown Siltstone Beds. The Gering sediments are a stratigraphic equivalent, of a part of the upper Sharps Formation [12] All of the evidence indicates the Brown Siltstone Beds are strongly associated with the Sharps Formation and the Nonpareil Ash Zone within the Brown Siltstone Beds is an extension of the Rockyford Ash Zone.

At the southernmost extension of the Brown Siltstone Beds in Nebraska, these beds are quite thin and overlain by 200+ feet of the Gering Formation. The Brown Siltstone Beds are thicker northward. At the Round Top to Adelia section a complete section of the Gering Formation displays a basal disconformity with the underlying Whitney Member of the Brule Formation. There is evidence of an Upper Whitney Ash (UWA) and an Upper Ashy Zone (UAZ). Proceeding to the northeast, the Gering Formation pinches out, the Whitney ash beds disappear, and the NPAZ appears at the base of the Brown Siltstone Beds [7] so that in the vicinity of the Chadron Arch and the town of White Clay, Nebraska, there is a fully developed section of the Brown Siltstone Beds with no overlying Gering Formation and no Whitney ashes (Figure 3). The NPAZ is a stratigraphic correlative of the RAZ, and the Brown Siltstone Beds in Nebraska occupy a position between the top of the Brule Formation and the Monroe Creek Formation identical to the Sharps Formation in South Dakota. Where the Gering Formation occurs in Nebraska, it disconformably overlies either Brule Formation sediments or the Brown Siltstone Beds. The Gering sediments are a stratigraphic equivalent, of a part of the upper Sharps Formation [12]. All of the evidence indicates the Brown Siltstone Beds are strongly associated with the Sharps Formation and the Nonpareil Ash Zone within the Brown Siltstone Beds is an extension of the Rockyford Ash Zone.

At the lower type section of the Sharps Formation, the Rockyford Ash immediately overlies sediments that weather similarly to Brule lithology. Ten miles north, at the type section of the Rockyford Member of the Sharps Formation is a single, thicker reworked ash layer with interbedded cut and fill structures slightly above Brule Formation sediments.

Twelve miles south of the Rockyford Member type section and five miles west of the lower type section of the Sharps Formation in the Grass Creek drainage system, there are two distinct ash layers at the base of the Sharps Formation separated by 31 feet. This corresponds to the stratigraphic position of the NPAZ of the Brown Siltstone Beds 20 miles south, in Nebraska, and to the RAZ at the lower type section of the Sharps Formation, and the ash layer on Sheep Mountain at the type section for the Rockyford Member of the Sharps Formation.

Between the upper portions of the Poleslide Member of the Brule Formation and the lower part of the Sharps Formation are massive siltstone deposits of varying thicknesses. Numerous local channel and overbank deposits occur above the Rockyford Member. The uppermost, persistent bedded deposit in the Sharps Formation, a unit consisting of 4 feet of red-brown thinly bedded claystone, occurs at 165 feet above the base of the Sharps Formation.

Also at 165 feet above the base of the Sharps Formation is a broad ranging area of silicified siltstone of varying thickness [4]. Some researchers report that Morris Skinner’s unpublished field notes refer to a “second white layer” at this level [7]. Tedford (1985) believed this layer to be the “probable equivalent of the Nonpareil Ash Zone” partially basing this supposition on paleomagnetic data available at the time [17] that later proved to be erroneous [15], [16]. More recent paleomagnetic studies show the Nebraska Nonpareil Ash Zone and the Rockyford Ash Zone to be equivalent, sharing a position at the base of Chron C11N [15], [16].

Approximately 169 feet above the base of the Sharps Formation massive siltstones were deposited that constitute the final 233 feet of the Sharps Formation.

The upper boundary of the Sharps Formation is comformable with the slope forming siltstones of the Sharps Formation and the massive vertical gray siltstone cliffs of the Monroe Creek Formation.

#### Composite Stratigraphic Column of the Sharps Formation

(Figure 4).

**Figure 4:**
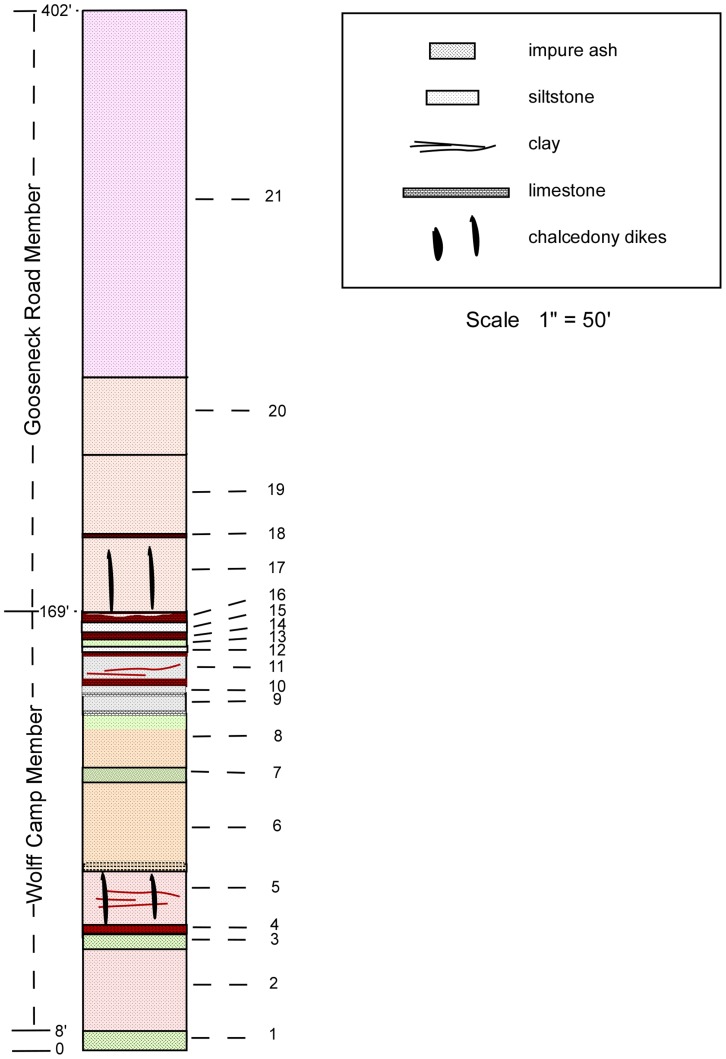
Composite Stratigraphic Column of the Sharps Formation. The Monroe Creek Formation forms vertical cliffs above slopes of the Sharps Formation. It weathers gray and presents as a massive, compact body of siltstone with no concretions. Sharps Formation (from top to bottom). 21 Silt, pinkish-tan, weathers to smooth steep slope; random vertically elongate light-tan nodules to 2 inches in diameter, 1–3 inches in length; nodules less abundant in upper 50 feet and almost absent in upper 20 feet; upper few feet grades into overlying Monroe Creek Formation. 115′; 20 Silt, tan, massive; small amount of very fine-grained sand. 28′; 19 Silt, tan, with randomly scattered vertically elongate nodules, 1–2 inches in diameter and 1–4 inches in length, more abundant upward. 60′; 18 Siltstone, red-brown, thinly bedded. 2′; 17 Silt, tan, with randomly scattered vertically elongate nodules, 1–2 inches in diameter and 1–4 inches in length; chalcedony and clastic dikes. 28′; 16 Claystone, red-brown, thinly bedded, with 2 thinly bedded clayey gray siltstone layers interspersed. 4′; 15 Siltstone, gray, massive 4′; 14 Siltstone, clayey, red-brown 3′; 13 Siltstone, greenish gray, sandy 3′; 12 Siltstone, gray 3′; 11 Siltstone, gray, with intermittent red-brown clayey layers; 1′ red-brown clayey siltstone at top, 3′ red-brown clayey layer at base. 12′; 10 Siltstone, gray. 3′; 9 Limestone; fresh surface shows scattered black dots; widespread but not continuous. May be multiple layers locally separated by gray siltstone. 8′; 8 Siltstone, tan with randomly scattered light-gray vertically elongate nodular concretions, 1–2 inches in diameter, 2–3 inches in length; upper 5′ massive with green tinge. 20′; 7 Sand, greenish gray; weathers white. 6′; 6 Silt, tan to light-brown, with vertically elongate nodular concretions, 1–4 inches in diameter, 2–6 inches in length. Two nodular layers at base. 34′; 5 Silt, somewhat clayey, pinkish gray; nodules that are sometimes traceable laterally as a layer; chalcedony and clastic dikes. 21′; 4 Claystone, reddish brown; lower 1.5′ stratified. 3′; 3 Ash with some silt, greenish white to white; upper 3′ clayey and stratified. 6′; 2 Silt, pinkish gray, clayey with 2″ red-brown nodular layer at base. 31′; 1 Ash with some silt, white to greenish white; upper portions stratified. 8′; **Total Sharps Formation thickness** **402 **ft. Brule Formation Siltstone, sandstone, and clay pinnacles; stair-steps as result of alternating layers; upper 20–30 feet contain several layers of siltstone and sandstone showing some steep cross-bedding.

**Figure 5:**
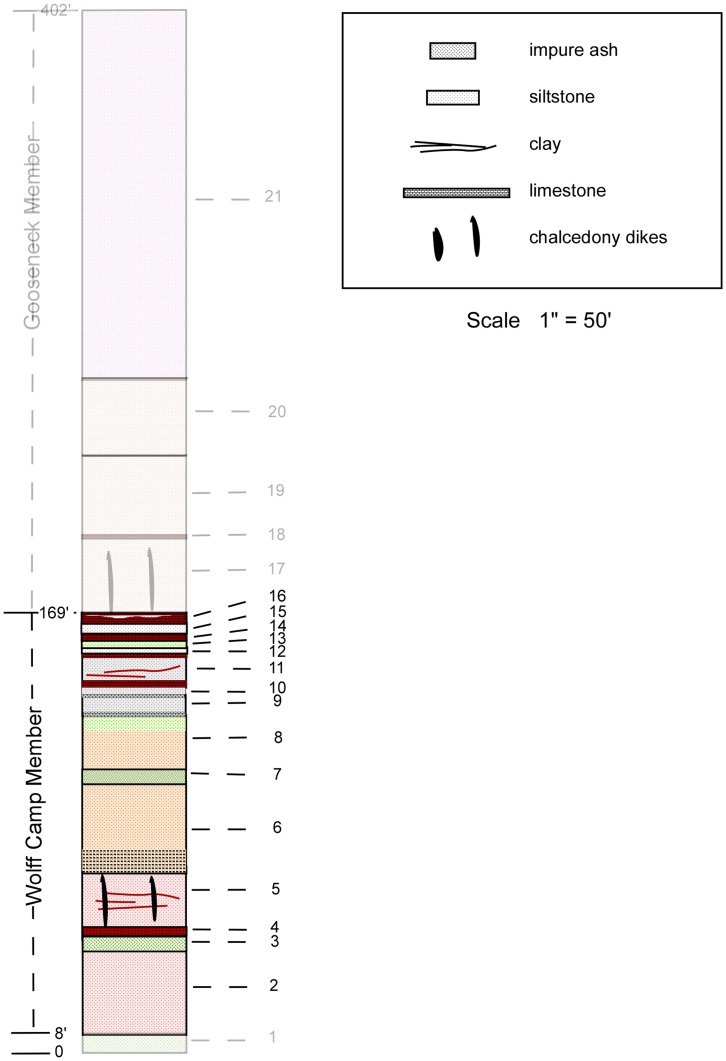
Wolff Camp Member of the Sharps Formation. Wolff Camp Member units (from top to bottom). 16 Claystone red-brown, thinly bedded, with 2 thinly bedded clayey gray siltstone layers interspersed. 4′; 15 Siltstone, gray, massive 4′; 14 Siltstone, clayey, red/brown 3′; 13 Siltstone, greenish gray, sandy 3′; 12 Siltstone, gray 3′; 11 Siltstone, gray, with intermittent red-brown clayey layers; 1′ red/brown clayey siltstone at top; 3′ red/brown clayey layer at base. 12′; 10 Siltstone, gray. 3′; 9 Limestone; fresh surface shows scattered black dots; widespread but not continuous. May be multiple layers locally separated by gray siltstone. 8′; 8 Siltstone, tan with randomly scattered light-gray vertically elongate nodular concretions, 1–2 inches in diameter, 2–3 inches in length; upper 5′ massive with green tinge. 20′; 7 Sand, greenish gray; weathers white. 6′; 6 Silt, tan to light-brown, with vertically elongate nodular concretions, 1–4 inches in diameter, 2–6 inches in length. Two nodular layers at base. 34′; 5 Silt, somewhat clayey, pinkish gray; a few nodules that are sometimes traceable laterally as a layer. 21′; 4 Claystone, reddish brown; lower 1.5′ stratified. 3′; 3 Ash with some silt, greenish white to white; upper 3′ clayey and stratified. 6′; 2 Silt, pinkish gray, clayey with 2″ red-brown nodular layer at base. 31′. Total Wolff Camp Member thickness **161** ft.

**Figure 6:**
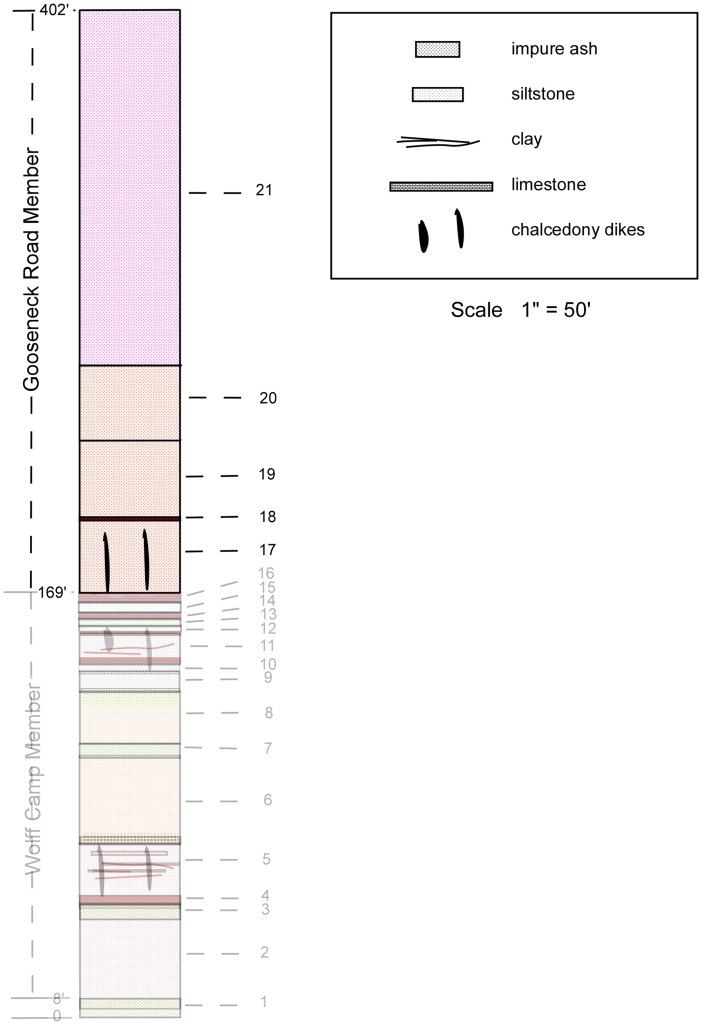
Gooseneck Road Member of the Sharps Formation. Gooseneck Road Member units (from top to bottom). 21 Silt, pinkish-tan, weathers to smooth steep slope; random vertically elongate light-tan nodules to 2 inches in diameter, 1–3 inches in length; nodules less abundant in upper 50 feet and almost absent in upper 20 feet; upper few feet grades into overlying Monroe Creek Formation. 115’; 20 Silt, tan, massive; small amount of very fine grained sand. 28′; 19 Silt, tan, with randomly scattered vertically elongate nodules, 1–2 inches in diameter and 1–4 inches in length, more abundant upward. 60′; 18 Siltstone, red-brown, thinly bedded. 2′; 17 Silt, tan, with randomly scattered vertically elongate nodules, 1–2 inches in diameter and 1–4 inches in length. Calcedony and clastic dikes. 28′. Gooseneck Road Member thickness **233 ft.**

The Monroe Creek Formation forms vertical cliffs above slopes of the Sharps Formation, weathers gray and presents as a massive, compact body of siltstone with no concretions.

Sharps Formation (from top to bottom).

21 Silt, pinkish-tan, weathers to smooth steep slope; random vertically elongate light-tan nodules to 2 inches in diameter, 1–3 inches in length; nodules less abundant in upper 50 feet and almost absent in upper 20 feet; upper few feet grades into overlying Monroe Creek Formation. 115’.
20 Silt, tan, massive; small amount of very fine-grained sand. 28′.
19 Silt, tan, with randomly scattered vertically elongate nodules, 1–2 inches in diameter and 1–4 inches in length, more abundant upward. 60′.
18 Siltstone, red-brown, thinly bedded. 2′.
17 Silt, tan, with randomly scattered vertically elongate nodules, 1–2 inches in diameter and 1–4 inches in length; chalcedony and clastic dikes. 28′.
16 Claystone, red-brown, thinly bedded, with 2 thinly bedded clayey gray siltstone layers interspersed. 4′.
15 Siltstone, gray, massive 4′.
14 Siltstone, clayey, red-brown 3′.
13 Siltstone, greenish gray, sandy 3′.
12 Siltstone, gray 3′.
11 Siltstone, gray, with intermittent red-brown clayey layers; 1 foot red-brown clayey siltstone at top, 3 foot red-brown clayey layer at base. 12′.
10 Siltstone, gray. 3′.
9 Limestone; fresh surface shows scattered black dots; widespread but not continuous; may be multiple layers locally separated by gray siltstone. 8′.
8 Siltstone, tan with randomly scattered light-gray vertically elongate nodular concretions, 1–2 inches in diameter, 2–3 inches in length; upper 5′ feet massive with green tinge. 20′.
7 Sand, greenish gray; weathers white. 6′.
6 Silt, tan to light-brown, with vertically elongate nodular concretions, 1–4 inches in diameter, 2–6 inches in length; two nodular layers at base. 34′.
5 Silt, somewhat clayey, pinkish gray; nodules that are sometimes traceable laterally as a layer; chalcedony and clastic dikes. 21′.
4 Claystone, reddish brown; lower 1.5 feet stratified. 3′.
3 Ash with some silt, greenish white to white; upper 3 feet clayey and stratified. 6′.
2 Silt, pinkish gray, clayey with 2inch red-brown nodular layer at base. 31′.
1 Ash with some silt, white to greenish white; upper portions stratified. 8′;

**Total Sharps Formation thickness 402 feet.**

### Brule Formation

Siltstone, sandstone, and clay pinnacles; stair-steps as result of alternating layers; upper 20–30 feet contain several layers of siltstone and sandstone showing some steep cross-bedding.

### Lithology of the Sharps Formation

The Sharps Formation has three distinct units, each bounded by distinct lithology:

- an upper expanse of massive siltstone
- a middle unit of floodplain/overbank, channel, and aeolian deposits, and
- the Rockyford Member, a reworked ash of highly variable thickness

We intend herein to name the two upper units.

### Rockyford Member

The Rockyford Member of the Sharps Formation [9] is an impure ash of variable thickness with gradational upper and lower contacts [13]. It seems to separate characteristic smooth slopes of overlying Sharps Formation from stairsteps and pinnacles of underlying Brule Formation [3].

At the type section of the Rockyford Member of the Sharps Formation on Sheep Mountain, moving up section from the Brule Formation, there is a gradual transition from Brule sediments and erosional features to the lithology of the Sharps Formation. Twenty to fifty feet above the gradational contact is the base of the Rockyford Ash. The Rockyford Ash is clearly the first ash layer moving up section from the Brule Formation. At the type section for the Rockyford Member of the Sharps Formation there is very little Tertiary material above the ash that could be used to place it in a proper stratigraphic context.

Similarly, at the lower type section of the Sharps Formation there is an ash at this position. However, at the lower type section and throughout most of the Pine Ridge Indian Reservation south of the White River, the column is not truncated immediately above the lowermost ash and there are two ash beds at the base of the Sharps Formation.

The lowermost ash layer will be referred to as the Rockyford Ash or the Rockyford Member of the Sharps Formation. The Rockyford Ash Zone (RAZ) is defined as the lower ash, the ash layer approximately 31 feet above it, as well as the intervening sediments. The Rockyford Ash Zone (RAZ) in South Dakota and the Nonpareil Ash Zone (NPAZ) in Nebraska share similarity in stratigraphic position, Radiometric dating, and positioning near the base of Chron C11N.

### Wolff Camp Member

Immediately overlying the Rockyford Member is 161 feet of floodplain/overbank, channel, and aeolian deposits we name the Wolff Camp Member of the Sharps Formation (Figure 5).

The derivation of this member is for the area near Sharps Corner, South Dakota, USA, known as Wolff Camp. Within this geographic area is the lower type section of the Sharps Formation that is also the type section for the Wolff Camp Member in the SW1/4 NW1/4 Sec 31, T41N, R42W Evergreen NE Quadrangle [3]. The stratification of the Wolff Camp Member, occurrence of fossils in nodules, numerous areas of lenticular siltstone, and deposition of reworked nodules are evidence of overbank/floodplain deposits. The particle size of the sediments is uniform and fine, suggesting fluvially reworked ashfall. The Wolff Camp Member is bounded by distinct lithology, the ashy complex of the Rockyford Member at the base and a 4 foot thick red-brown clayey siltstone that marks the upper limit of the widespread floodplain/overbank and channel bedded deposits of the Wolff Camp Member.

#### Composite Stratigraphic column of Wolff Camp Member

(Figure 5).

Wolff Camp Member units (from top to bottom).

16 Claystone red-brown, thinly bedded, with 2 thinly bedded clayey gray siltstone layers interspersed. 4′.
15 Siltstone, gray, massive 4′.
14 Siltstone, clayey, red/brown 3′.
13 Siltstone, greenish gray, sandy 3′.
12 Siltstone, gray 3′.
11 Siltstone, gray, with intermittent red-brown clayey layers; 1 foot red/brown clayey siltstone at top; 3 foot red/brown clayey layer at base. 12′.
10 Siltstone, gray. 3′.
9 Limestone; fresh surface shows scattered black dots; widespread but not continuous; may be multiple layers locally separated by gray siltstone. 8′.
8 Siltstone, tan with randomly scattered light-gray vertically elongate nodular concretions, 1–2 inches in diameter, 2–3 inches in length; upper 5 feet massive with green tinge. 20′.
7 Sand, greenish gray; weathers white. 6′.
6 Silt, tan to light-brown, with vertically elongate nodular concretions, 1–4 inches in diameter, 2–6 inches in length. Two nodular layers at base. 34′.
5 Silt, somewhat clayey, pinkish gray; a few nodules that are sometimes traceable laterally as a layer. 21′.
4 Claystone, reddish brown; lower 1.5 feet stratified. 3′.
3 Ash with some silt, greenish white to white; upper 3 feet clayey and stratified. 6′.
2 Silt, pinkish gray, clayey with 2″ red-brown nodular layer at base. 31′;

Wolff Camp Member thickness 161 feet.

### Gooseneck Road Member

The approximately 233 feet of massive siltstone from the top of the Wolff Camp Member to the base of the superjacent Monroe Creek Formation we name the Gooseneck Road Member of the Sharps Formation (Figure 6). The type section is at the crest of the divide between Porcupine Creek and Wounded Knee Creek on Gooseneck Road. The type section for the Gooseneck Road Member is the same as that for the upper type section of the Sharps Formation at NW1/4 Sec 30 to NE1/4 Sec 20, T39N, R43W [3]. The Gooseneck Road Member is generally devoid of any widespread bedding.

#### Composite Stratigraphic Column of Gooseneck Road Member

(Figure 6).

Gooseneck Road Member units (from top to bottom).

21 Silt, pinkish-tan, weathers to smooth steep slope; random vertically elongate light-tan nodules to 2 inches in diameter, 1–3 inches in length; nodules less abundant in upper 50 feet and almost absent in upper 20 feet; upper few feet grades into overlying Monroe Creek Formation. 115’.
20 Silt, tan, massive; small amount of very fine grained sand. 28′.
19 Silt, tan, with randomly scattered vertically elongate nodules, 1–2 inches in diameter and 1–4 inches in length, more abundant upward. 60′.
18 Siltstone, red-brown, thinly bedded. 2′.
17 Silt, tan, with randomly scattered vertically elongate nodules, 1–2 inches in diameter and 1–4 inches in length. Calcedony and clastic dikes. 28′;

Gooseneck Road Member thickness 233 feet.

### Brown Siltstone Beds

Those unnamed sediments above the unconformity at the top of the Brule Formation and below the unconformity at the base of the Gering Formation, in Nebraska, informally recognized as the Brown Siltstone [11], [12] should be elevated to a member status within the Sharps Formation. LaGarry (1998) revised the description of the Brule Formation of Nebraska and informally referred to the Brown Siltstone Beds as the ‘horn member’ of the Brule Formation, deferring to Swinehart for formal naming. Through LaGarry’s work and the subsurface analysis of Swinehart, sediments previously called Brown Siltstone Beds can be recognized as a southern continuation of the Sharps Formation equivalent to the Rockyford Member and the Wolf Camp Member of the Sharps Formation. Paleomagnetic and lithologic consistencies across the Chadron Arch support the recognition of this correlation and will be an added datum assisting researchers in future biostratigraphic studies (Figure 2).

Formal naming of the Brown Siltstone Beds as a member of the Brule Formation would further confuse the stratigraphy of the Great Plains. We propose an interim informal designation of Horn Member of the Sharps Formation. In lieu of formal naming of the member, we also defer to the original researchers with the hope that they too will appreciate the ongoing findings of Sharps Formation correlation.

[Note: The original assignment of the Sharps Formation to the Arikaree Group was not representative of the similarity of the lithology of the Sharps Formation to White River Group lithology. This erroneous assignment has hampered the correlation of the Sharps Formation sediments to other White River Group sediments. The Sharps Formation sediments display lithology characteristic of the White River Group and should be reclassified to the White River Group from the overlying Arikaree Group.].
